# Intravital imaging of mouse urothelium reveals activation of extracellular signal‐regulated kinase by stretch‐induced intravesical release of ATP

**DOI:** 10.14814/phy2.13033

**Published:** 2016-11-15

**Authors:** Takeshi Sano, Takashi Kobayashi, Hiromitsu Negoro, Atsushi Sengiku, Takuya Hiratsuka, Yuji Kamioka, Louis S. Liou, Osamu Ogawa, Michiyuki Matsuda

**Affiliations:** ^1^ Department of Pathology and Biology of Diseases Graduate School of Medicine Kyoto University Kyoto Japan; ^2^ Department of Urology Graduate School of Medicine Kyoto University Kyoto Japan; ^3^ Department of Molecular Genetics Institute of Biomedical Science Kansai Medical University Osaka Japan; ^4^ Department of Urology Cambridge Health Alliance Cambridge Massachusetts

**Keywords:** Extracellular signal‐regulated kinase, in vivo imaging, urothelium

## Abstract

To better understand the roles played by signaling molecules in the bladder, we established a protocol of intravital imaging of the bladder of mice expressing a Förster/fluorescence resonance energy transfer (FRET) biosensor for extracellular signal‐regulated kinase (ERK), which plays critical roles not only in cell growth but also stress responses. With an upright two‐photon excitation microscope and a vacuum‐stabilized imaging window, cellular ERK activity was visualized in the whole bladder wall, from adventitia to urothelium. We found that bladder distention caused by elevated intravesical pressure (IVP) activated ERK in the urothelium, but not in the detrusor smooth muscle. When bladder distension was prevented, high IVP failed to activate ERK, suggesting that mechanical stretch, but not the high IVP, caused ERK activation. To delineate its molecular mechanism, the stretch‐induced ERK activation was reproduced in an hTERT‐immortalized human urothelial cell line (TRT‐HU1) in vitro. We found that uniaxial stretch raised the ATP concentration in the culture medium and that inhibition of ATP signaling by apyrase or suramin suppressed the stretch‐induced ERK activation in TRT‐HU1 cells. In agreement with this in vitro observation, pretreatment with apyrase or suramin suppressed the high IVP‐induced urothelial ERK activation in vivo. Thus, we propose that mechanical stretch induces intravesical secretion of ATP and thereby activates ERK in the urothelium. Our method of intravital imaging of the bladder of FRET biosensor‐expressing mice should open a pathway for the future association of physiological stimuli with the activities of intracellular signaling networks.

## Introduction

To improve understanding of the physiological responses of the urothelium, several research groups have explored in vivo or ex vivo microscopy of rodent bladders. Koenig et al. (1999) used confocal laser‐scanning microscopy to observe the rat urothelium after cystotomy. More recently, two‐photon excitation microscopy (2PM) has been applied to observe mouse bladders ex vivo (Zhuo et al. 2009; Schueth et al. 2014). However, presumably due to the difficulties associated with fixation of the bladder and light scattering caused by the thick muscular layer, less invasive live imaging of the bladder has not been achieved.

Another reason why intravital bladder imaging has not been realized may be that, without improved functional or molecular information about the urothelium, live imaging of the urothelium per se may not be expected to yield novel findings. To meet the demand for new molecular and functional information, one possible approach would be using biosensors based on the principle of Förster/fluorescence resonance energy transfer (FRET). FRET is a process by which a donor fluorophore in an excited state transfers energy to a neighboring acceptor fluorophore, thereby causing the acceptor to emit fluorescence at its characteristic wavelength. A number of GFP‐based FRET biosensors have already been used to study the spatio‐temporal regulation of signaling molecules in tissue culture cells (Aoki et al. 2013a; Oldach and Zhang 2014; Enterina et al. 2015; Miyawaki and Niino 2015). The recent development of transgenic mice expressing bright FRET biosensors, called FRET mice, has further paved the way to the observation of molecular activities in live tissues (Kamioka et al. 2012). For example, we and others have reported activation of extracellular signal‐regulated kinase (ERK) during extravasation of neutrophils, activation of ERK in melanoma cells after anti‐BRaf drug administration, and Rac1 activation during mouse embryonic development (Johnsson et al. 2014; Mizuno et al. 2014; Hirata et al. 2015).

Mechanical stress to the urothelium has been shown to trigger ATP release to both the luminal and basal side (Ferguson et al. 1997; Lewis and Lewis 2006; Mochizuki et al. 2009; Miyamoto et al. 2014; Beckel et al. 2015). ATP released to the basal side activates purinergic receptors such as P2X_2_ and P2X_3_ on the afferent nerves in the suburothelium (Cockayne et al. 2000, 2005; Vlaskovska et al. 2001; Rong et al. 2002; Yu and de Groat 2008). On the other hand, ATP released to the luminal side has been reported to stimulate suburothelial C‐fibers only under pathological conditions, due to low permeability of the urothelium (Pandita and Andersson 2002; Nishiguchi et al. 2005). More recently, however, it has been reported that ATP secreted from the urothelium to the luminal side via Pannexin 1 activates urothelial P2X and P2Y receptors in an autocrine/paracrine manner and thereby modulates the voiding function, suggesting a positive feedback loop of ATP secretion (Negoro et al. 2014; Beckel et al. 2015).

The aim of this study was to fill the gap between cell biological studies in vitro and physiological observations in vivo, focusing on the response of urothelial cells under increased intravesical pressure (IVP) and accompanying mechanical stretch. Since ATP signaling initiated by mechanical stress and injury has been shown to activate ERK in various tissues (Neary et al. 2003; Yin et al. 2007; Okumura et al. 2008; Ito et al. 2014), albeit not in the bladder, we monitored ERK activity in FRET mice expressing the biosensor for ERK (Kamioka et al. 2012). We observed robust and rapid ERK activation upon application of high IVP. Further experiments have suggested that the ERK activation was dependent on the increased stretch, but not the elevated hydrostatic pressure. Thus, the live imaging of FRET mice is expected to create a new bridge between cell biology and physiology.

## Methods

### Ethical approval

The animal protocols were reviewed and approved by the Animal Care and Use Committee of Kyoto University Graduate School of Medicine (nos. 12064, 13074, 14079, and 15064).

### Animals

**Figure 1:**
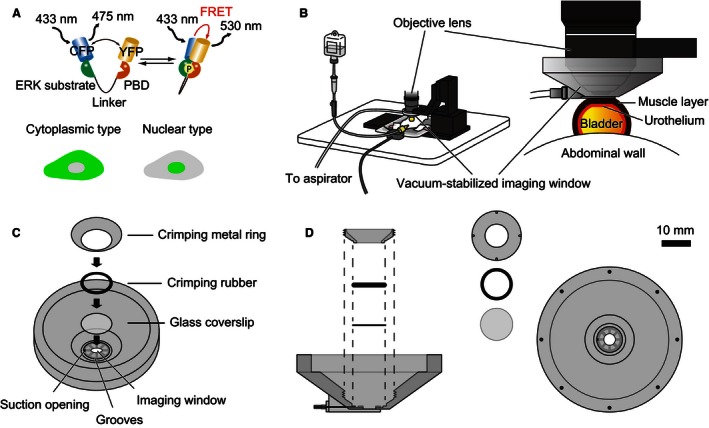
Experimental set‐up for intravital imaging of the mouse bladder. (A) The mode of action of a Förster/fluorescence resonance energy transfer (FRET) biosensor for extracellular signal‐regulated kinase (ERK) activity, EKAREV. Phosphorylation of the ERK substrate region within the biosensor induces conformational change, which brings yellow fluorescent protein (YFP) into close proximity of cyan fluorescent protein (CFP) and YFP, and thereby increases the level of FRET. The phosphate binding domain binds to the ERK substrate region in a phosphorylation‐dependent manner. Two types of biosensors, the EKAREV‐nuclear export signal and EKAREV‐nuclear localization signal, are in the cytoplasm and the nucleus, respectively. (B) Layout of the intravital imaging system for the mouse bladder. An anesthetized mouse is placed on an electric heat pad. The bladder is attached to the vacuum‐stabilized imaging window. (C) Detailed schemes of the vacuum‐stabilized imaging window. The bowl of the imaging window, which has a glass coverslip at the bottom, is designed to be filled with water for the water‐immersion objective. The suction opening is placed on the bottom surface of the imaging window to enable the aspirator to continuously suck on the bladder. (D) The side and top views of the vacuum‐stabilized imaging window. The crimping metal ring and a crimping rubber are used to seal the water reservoir. The crimping metal ring has four small pits to fit a special screwdriver.

Transgenic mice expressing ERK FRET biosensors and the negative control FRET biosensor, AKAR3EV‐NC, have been described previously (Kamioka et al. 2012). Two ERK FRET biosensors, namely, the EKAREV‐nuclear export signal (NES) and EKAREV‐nuclear localization signal (NLS), are in the cytoplasm and the nucleus, respectively (Fig. 1A) (Kamioka et al. 2012). Founder mice of EKAREV‐NES and EKAREV‐NLS were backcrossed more than five generations to C57BL/6N Jcl before analysis. The resulting mice expressing EKAREV‐NES and EKAREV‐NLS are called Eisuke‐NES mice and Eisuke‐NLS mice, respectively. Founder mice expressing EKAREV‐NLS were also backcrossed to FVB/N Jcl to yield Eisuke‐NLS‐FVB mice. Mice were housed in a specific pathogen‐free facility in temperature‐controlled rooms with a 12‐h light/12‐h dark cycle and received a routine chow diet and water ad libitum. To date, no disease or anomaly has been associated with the expression of the FRET biosensors used in this study. For intravital imaging and experiments with animal tissue, 8–30‐week‐old female mice were used. Mice were anesthetized through the inhalation of 1–1.5% isoflurane (Abbott Laboratories, North Chicago, IL).

### 2PM and image processing

We used an FV1000MPE‐BX61WI upright microscope (Olympus, Tokyo, Japan) equipped with a 25×/1.05 water‐immersion objective lens (XLPLN 25XWMP; Olympus), and an InSight DeepSee Ultrafast laser (0.95 W at 900 nm; Spectra Physics, Mountain View, CA). The excitation wavelength for cyan fluorescent protein (CFP) was 840 nm. An IR‐cut filter, BA685RIF‐3, two dichroic mirrors, DM505 and DM570, and four emission filters, FF01‐425/30 (Semrock, Rochester, NY) for the second harmonic generation (SHG), BA460‐500 (Olympus) for CFP, BA520‐560 (Olympus) for yellow fluorescent protein (YFP), and 645/60 (Chroma Technology Corp., Bellows Falls, VT) for Qtracker 655 (Life Technologies, Carlsbad, CA), were used. The microscope was equipped with a two‐channel GaAsP detector unit and two built‐in photomultiplier tubes. FluoView software (Olympus) was used to control the microscope and to acquire images, which were saved in the multilayer 16‐bit Tagged Image File Format. Recorded images were processed and analyzed with MetaMorph software (Molecular Devices, Sunnyvale, CA).

### Preparation of mice for intravital imaging

The anesthetized mice were placed in the supine position on an electric heat pad maintained at 37°C. The hind legs of the mice were fixed to the heat pad with adhesive tape. A 24‐gauge ethylene tetrafluoroethylene catheter (Terumo Corporation, Tokyo, Japan) connected to a 50 mL bottle of normal saline (Otsuka Pharmaceutical Factory Inc., Tokushima, Japan) was inserted transurethrally into the bladder. The urethra was ligated using 5‐0 silk surgical sutures (Nesco Suture; Alfresa Pharma Corporation, Osaka, Japan) to prevent urine leakages. The IVP was controlled by the bottle's height. The bladder was pulled out of the abdominal cavity through an abdominal incision, and the anterior to cephalad bladder wall was immobilized on a custom‐made vacuum‐stabilized imaging window (Olympus Engineering Corporation, Tokyo, Japan), which was connected to a vacuum aspirator and designed to continuously pull on the bladder wall (Fig. 1B). The suction force was set to approximately 20 kPa, and the blood flow in the bladder was not detectably affected under this condition. The bowl of the imaging window was warmed to 37.5°C with a lens heater (TP‐LH; Tokai Hit, Shizuoka, Japan). At the end of the experiments, euthanasia was performed by anesthetic overdose.

### Intravital multidimensional imaging of the whole bladder wall

Intravital imaging of mouse intestines using 2PM was performed as described previously (Mizuno et al. 2014). Z‐stack images of the whole bladder wall of Eisuke‐NES and Eisuke‐NLS mice were acquired from the urothelium to the adventitia at 0.5 or 1.0 *μ*m intervals and at a scan speed of 4 *μ*s per pixel. The laser power was adjusted using the Bright Z mode to obtain similar signal intensities from the cells in each plane. CFP, Qtracker 655 and SHG were imaged to show cells, blood vessels and collagen fibers, respectively.

### Visualization and quantification of ERK activity in urothelial cells of live mice

We used Eisuke‐NLS‐FVB and Eisuke‐NLS mice to visualize ERK activity in urothelial cells and smooth muscle cells. Images were acquired every 3 or 5 min using a 1.4–1.6 digital zoom at a scan speed of 4 *μ*s per pixel. The FRET/CFP ratio image was prepared to show ERK activity as described previously (Mizuno et al. 2014). In brief, the FRET level is represented by the FRET/CFP ratio image in the intensity‐modulated display mode. Eight colors from red to blue represent the FRET/CFP ratio, and the 32 grades of color intensity represent the signal intensity of the CFP image. The warm and cold colors indicate high and low FRET levels, respectively. The FRET/CFP ratio was quantified for each cell as follows. A region of interest was set to include each nucleus on a pair of background‐subtracted CFP and FRET images. The average fluorescence intensity of the region of interest was calculated for both CFP and FRET images and used to calculate the FRET/CFP ratio of each cell. This FRET/CFP ratio was used to represent ERK activity. All imaging experiments began approximately 1 h after transurethral catheterization, when the ERK activity became stable. The IVP was maintained at 15–20 cm H_2_O, unless high IVP overload was applied. For the high IVP condition, the IVP was increased from 15 to 20 cm H_2_O to 60 or 100 cm H_2_O by changing the height of the bottle of saline.

### Administration of drugs to live mice

For systemic administration, 200 *μ*L PBS containing 4 *μ*L Qtracker 655 alone or 4 *μ*L Qtracker 655 and PD0325901 (5 mg kg^−1^), a mitogen‐activated protein kinase/ERK kinase (MEK) inhibitor (EMD Millipore, Billerica, MA), was intravenously injected. For local administration into the bladder lumen, apyrase and suramin diluted in 100 *μ*L PBS (37°C) was transurethrally injected through a 24‐gauge catheter.

### Platelet velocity profiles in suburothelial arterioles

To assess the microhemodynamics, platelet velocity profiles were examined in the suburothelial arterioles, the diameters of which were between 15 and 30 *μ*m. Using a 25×/1.05 water‐immersion objective lens, a viewfield of 84.8 × 84.8 *μ*m (320 × 320 pixels, 0.265 *μ*m per pixel) was scanned at 2 *μ*s per pixel (1.8 frames sec^−1^). Each line scan took 1.736 ms, which is the sum of 0.640 ms (2 *μ*s per pixel multiplied by 320 pixels) and 1.096 ms (interval between the lines). Under these imaging conditions, the platelets were recorded as dotted tracks. The stroboscopic measurement of the platelets enabled us to determine the velocity of each platelet as described below.

The time lag between coordinates *a* (*Xa*,* Ya*) and *b* (*Xb*,* Yb*) was given by the following equation:

$$\text{Time}\;\text{lag}\;\left(\text{sec}\right)=2\times 10^{-6}\times \left(Xb-Xa\right)+1.736\times 10^{-3}\times \left(Yb-Ya\right)$$

The distance between the coordinates was as follows:

$$\text{Distance}\;\left(\text{mm}\right)=0.265\times 10^{-3}\times \sqrt{{\left(Xb-Xa\right)}^{2}+{\left(Yb-Ya\right)}^{2}}$$

Therefore, the platelet velocity was determined using the following equation:

$$\text{Velocity}\;\left(\text{mm}\;{\text{sec}}^{-1}\right)=0.265\times 10^{-3}\times \sqrt{{\left(Xb-Xa\right)}^{2}+{\left(Yb-Ya\right)}^{2}}/\left\{2\times 10^{-6}\times \left(Xb-Xa\right)+1.736\times 10^{-3}\times \left(Yb-Ya\right)\right\}$$

### Solutions and reagents

ATP, apyrase, suramin, and epidermal growth factor (EGF) were purchased from Sigma‐Aldrich (St. Louis, MO). Capsaicin, 4*α*‐phorbol 12,13‐didecanoate (4*α*‐PDD) and carbachol were purchased from Wako Pure Chemical (Osaka, Japan). Trametinib was purchased from LC Laboratories (Woburn, MA). Pyridoxalphosphate‐6‐azophenyl‐2′,4′‐disulfonic acid (PPADS) was purchased from Abcam (Cambridge, MA). AG1478 was purchased from BioVision Inc. (Milpitas, CA).

### Cell culture and establishment of a stable cell line

HEK‐293T cells were maintained in Dulbecco's modified Eagle's medium (DMEM; Thermo Fischer Scientific, Waltham, MA), supplemented with 10% heat‐inactivated fetal bovine serum (FBS; Sigma‐Aldrich) and penicillin/streptomycin (Nacalai Tesque, Kyoto, Japan). The hTERT‐immortalized human urothelial cell line (TRT‐HU1) was a gift from Rosalyn M. Adam (Urological Diseases Research Center, Boston Children's Hospital, Boston, MA). TRT‐HU1 cells were maintained in DMEM containing 15% heat‐inactivated FBS, 2 mmol L^−1^ l‐glutamine, 110 mg L^−1^ sodium pyruvate, nonessential amino acids (Life Technologies), 1.15 mmol L^−1^ 1‐thioglycerol and penicillin/streptomycin (Kim et al. 2011). Both TRT‐HU1 and HEK‐293T cells were incubated in a humidified atmosphere of 5% CO_2_ in air at 37°C. For lentiviral production, HEK‐293T cells were cotransfected with the pCSII‐EF vector encoding the FRET biosensor for ERK, psPAX2 (plasmid #12260; Addgene, Cambridge, MA) and pCMV‐VSV‐G‐RSV‐Rev (RIKEN BioResource Center, Tsukuba, Japan). Virus‐containing media were filtered and collected at 48 h after transfection. The target cells were infected in the presence of 10 *μ*g mL^−1^ polybrene (Nacalai Tesque). Two days after infection, the infected cells were selected with 15 *μ*g mL^−1^ puromycin (Sigma‐Aldrich).

### Time‐lapse FRET imaging of TRT‐HU1 cells

FRET imaging of tissue culture cells was performed as previously reported (Aoki and Matsuda 2009). TRT‐HU1 cells expressing FRET biosensors were starved for 2–3 h with phenol red‐free Medium 199 (Life Technologies). After 30 min of imaging, cells were treated with ATP or EGF, or were subjected to uniaxial stretch or hydrostatic pressure. Cells were imaged with an inverted fluorescence microscope (IX83; Olympus) equipped with a 20× objective lens (Olympus), a cooled CCD camera (CoolSNAP HQ or CoolSNAP K4; Roper Scientific, Tucson, AZ), an LED illumination system (CoolLED precisExcite; Molecular Devices), an IX2‐ZDC laser‐based autofocusing system (Olympus), and an MD‐XY30100T‐Meta automatically programmable XY stage (SIGMA KOKI, Tokyo, Japan). The following filters used for the dual‐emission imaging studies were obtained from Omega Optical (Brattleboro, VT): an XF1071 (440AF21) excitation filter, an XF2034 (455DRLP) dichroic mirror, and two emission filters (XF3075 480AF30 for CFP and XF3079 535AF26 for YFP).

### Application of hydrostatic pressure to TRT‐HU1 cells

A three‐way stopcock was installed to a 4 mm‐diameter hole made in the lid of a 35‐mm glass‐based dish (AGC Techno Glass, Shizuoka, Japan). To the stopcock, a bottle of Medium 199 was connected via an infusion set (Terumo Corporation). A sampling window was also prepared in the lid to manipulate the medium. The medium bottle was set up at a height of 60 cm for loading hydrostatic pressure.

### Mechanical stretch experiment on TRT‐HU1 cells

An elastic silicone chamber (STB‐CH‐04; STREX, Osaka, Japan) was used for the stretch experiments. The silicon chamber was autoclaved in distilled water at 121°C for 20 min, and was coated with 10 *μ*g mL^−1^ of fibronectin (Sigma‐Aldrich). The silicon chamber containing confluent TRT‐HU1 cells and 500 *μ*L culture medium was attached to the manual extension device (STB‐10‐04; STREX) and was set up on the microscope stage. A 10‐mm stretch of the chamber, which induced 50% uniaxial stretch of the cells, was applied at a speed of 0.4 mm sec^−1^.

### Western blotting

The urinary bladder of anesthetized mice was excised, immediately sunk into PBS cooled with ice, and cut open longitudinally. Then the urothelium was gently separated from the underlying tissue using fine forceps and scissors under a dissecting microscope. The separated urothelium was lysed in SDS sample buffer (62.5 mmol L^−1^ Tris‐HCl [pH 6.8], 12% glycerol, 2% SDS, 0.004% bromophenol blue and 5% 2‐mercaptoethanol). After sonication and boiling at 95°C, the samples were resolved by SDS‐PAGE on SuperSep Ace 5–20% precast gels (Wako Pure Chemical), and transferred to PVDF membranes (Merck Millipore, Darmstadt, Germany). After 60‐min incubation with Odyssey blocking buffer (LI‐COR Biosciences, Lincoln, NE) at room temperature, the membranes were incubated for 2 h at 4°C with mouse monoclonal anti‐phospho‐ERK1/2 antibodies (1:2,000; Cell Signaling Technology, Danvers, MA) and rabbit monoclonal anti‐ERK1/2 antibodies (1:2,000; Cell Signaling Technology). The immunoreactivities were visualized with IRDye 800CW‐conjugated donkey anti‐mouse IgG antibodies (1:15,000; LI‐COR Biosciences) and IRDye 680LT‐conjugated goat anti‐rabbit IgG antibodies (1:15,000; LI‐COR Biosciences). All antibodies were diluted in Odyssey blocking buffer. Proteins were detected by an Odyssey Infrared Imaging System (LI‐COR Biosciences) and analyzed using the Odyssey imaging software.

### Measurement of ATP release

The luciferin‐luciferase assay (CellTiter‐Glo Luminescent Cell Viability Assay; Promega, Madison, WI) was used according to the manufacturer's protocol to quantify the amounts of ATP released in the bladder lumen and in the bathing solution of TRT‐HU1 cells. Briefly, 20 *μ*L samples taken from the bladder of anesthetized mice and the bathing solution of TRT‐HU1 cells were individually placed in white‐walled 96‐well plates (Nunc F96 MicroWell; Thermo Fischer Scientific), and 100 *μ*L CellTiter‐Glo reagent was added directly to each well. The plates were then incubated at room temperature for 15 min on a shaker, and transferred to the Multilabel Plate Reader VICTOR X5 (PerkinElmer, Waltham, MA), where luminescence was measured using a 1 sec integration time. The ATP concentration in the samples was calculated from standard curves constructed using ATP from 20 nmol L^−1^ to 20 *μ*mol L^−1^.

### Statistical analysis

Data are shown as the mean ± SD, unless otherwise indicated. For two‐group comparisons, we used paired or unpaired Student's *t*‐tests. For comparing more than two groups, differences between groups were tested for significance using one‐way ANOVA. For post hoc comparisons with the control group, Dunnett's test was used. Although some of the data are expressed as the fold change from the control, all statistical analyses were performed on raw data. All statistical analyses were performed using GraphPad Prism5 software (GraphPad Software Inc., La Jolla, CA). *P* values < 0.05 were considered statistically significant.

## Results

### 2PM visualizes the whole mouse bladder wall in live mice

In order to perform intravital imaging of the mouse bladder wall using 2PM, two conditions must be met. First, motion artifacts caused by the animal's heart beating and breathing have to be minimized. This task was achieved using a custom‐made vacuum‐stabilized imaging window (Fig. 1B–D). Second, because the urothelium will be visualized through the thick bladder wall, the resultant light scattering must be overcome. We solved this problem by applying 15–20‐cm H_2_O IVP, which distended the bladder to reduce wall thickness to 150–250 *μ*m (Fig. 1B). Of note, IVP in the physiological urine storage state ranges from 5 to 10 cm H_2_O (Takezawa et al. 2014; Yu et al. 2014).

**Figure 2:**
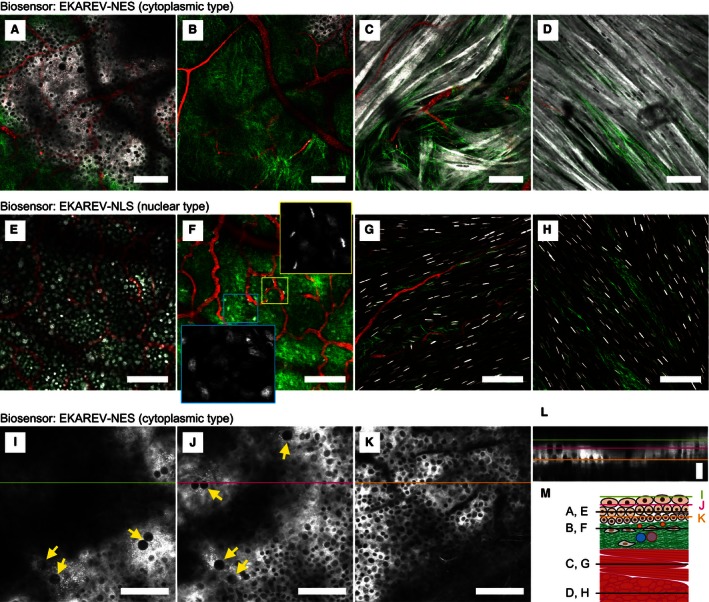
Multidimensional imaging of the whole bladder wall of live mouse by two‐photon excitation microscopy. (A–H) Merged images of cyan fluorescent protein (CFP) (gray scale), second harmonic generation (green) and Qtracker 655 (red) by multidimensional imaging of the whole mouse bladder wall expressing EKAREV‐NES (A–D, and Video S1) and EKAREV‐NLS (E–H, and Video S2). Magnified images of the boxed regions show only CFP (gray scale). (I–K) Multidimensional CFP images (gray scale) of the urothelium expressing EKAREV‐NES with a 1.4 digital zoom. Umbrella cells with large nuclei are indicated by arrows. Scale bar = 100 *μ*m (A–K). (L) A reconstructed *xz*‐plane image at the lines drawn in I–K. The image is extended along the *z*‐axis to 240% for better visibility. The colors of bars indicating the imaging planes correspond to those in I–K. Scale bar = 10 *μ*m. (M) A schematic diagram of the whole bladder wall with each imaging plane indicated.

First, we show the whole bladder wall of a live transgenic mouse expressing the cytoplasmic FRET biosensor, EKAREV‐NES (Eisuke‐NES mouse; Video S1). The cytoplasmic distribution of the FRET biosensor allowed us to distinguish cell types by the shape and location of each cell (Fig. 2A–D). SHG and the intravenous administration of Qtracker 655 visualized collagen fibers and suburothelial capillaries in the lamina propria, respectively (Fig. 2B). The muscularis propria, a detrusor smooth muscle layer, contained muscle cells with branched cell bodies (Fig. 2C and D). The smooth muscle exhibited rhythmic contractions (Video S1). At least two layers were distinguishable because of differences in the muscle fiber arrays between the layers (Fig. 2C and D). Whole bladder images were also obtained using a transgenic mouse expressing the nuclear‐type FRET biosensor, EKAREV‐NLS (Eisuke‐NLS mouse; Fig. 2E–H, and Video S2).

Identification of cell types was based on the shape and location of individual cells. For example, interstitial cells were identified by an elliptic nucleus just under the urothelium and endothelial cells were identified by a slender nucleus along an arteriole as shown in the magnified images (Fig. 2F). Within the urothelium, umbrella cells, intermediate cells, and basal cells were distinguished from each other by their location and the size of their nuclei (Fig. 2I–L). A schematic diagram of the whole bladder wall indicates each imaging plane (Fig. 2M). In the following experiments, we used the nuclear‐type FRET biosensor because it allows easier quantification of ERK activity in each cell than the cytoplasmic‐type FRET biosensor. At least in the case of EKAREV, we have not detected a significant difference in the FRET/CFP ratio between the cytoplasmic‐ and nuclear‐type biosensors (Kamioka et al. 2012).

### Bladder distention with increased IVP activates ERK in the urothelium

**Figure 3:**
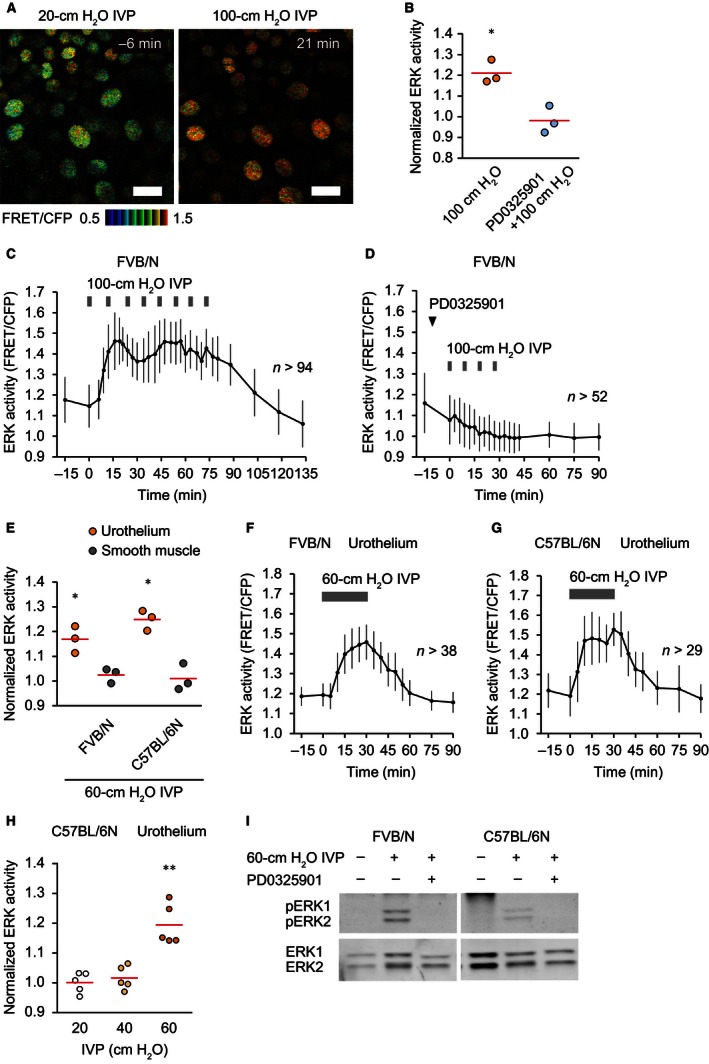
Extracellular signal‐regulated kinase (ERK) activation by bladder distention with increased intravesical pressure (IVP) in the urothelium. (A) Representative ratio images of high IVP‐induced ERK activation in the urothelium of an Eisuke‐NLS‐FVB mouse (Video S3). Scale bar = 20 *μ*m. (B) Summary of the relative change in urothelial ERK activity of Eisuke‐NLS‐FVB mice by intermittent 1‐min 100‐cm H_2_O IVP and the effect of an MEK inhibitor, PD0325901 (5 mg kg^−1^), on high IVP‐induced ERK activation (*n *=* *3 for each). ERK activity was measured at baseline (0 min) and 15 min after application of the first 1‐min of 100‐cm H_2_O IVP. PD0325901 was injected intravenously 10 min before application of high IVP. Circles and red lines indicate normalized ERK activity in each experiment and the mean values, respectively. (C and D) Representative graphs of the change in urothelial ERK activity by intermittent 1‐min 100‐cm H_2_O IVP (C) and the effect of PD0325901 on high IVP‐induced ERK activation (D). *n*, number of cells analyzed at each time point. (E) Summary of the relative change in ERK activity by 30‐min 60‐cm H_2_O IVP in the urothelium and the smooth muscle of both Eisuke‐NLS‐FVB and Eisuke‐NLS mice (*n *=* *3 for each). ERK activity was measured at baseline (0 min) and 30 min after application of high IVP. Circles and red lines indicate normalized ERK activity in each experiment and the mean values, respectively. (F and G) Representative graphs of the change in urothelial ERK activity of Eisuke‐NLS‐FVB (F) and Eisuke‐NLS mice (G) by 30‐min 60‐cm H_2_O IVP. *n*, number of cells analyzed at each time point. (H) Summary of the relative change in urothelial ERK activity of Eisuke‐NLS mice by 30‐min 20‐, 40‐, or 60‐cm H_2_O IVP (*n *=* *5 for each). ERK activity was measured at baseline (0 min) and 30 min after application of IVP. Circles and red lines indicate normalized ERK activity in each experiment and the mean values, respectively. (I) Western blot analysis showing ERK1/2 and phospho‐ERK1/2 in the isolated urothelium of Eisuke‐NLS‐FVB and Eisuke‐NLS mice. Bladders were extracted without application of high IVP (left lane), after application of 30‐min 60‐cm H_2_O IVP (middle lane) and after application of 30‐min 60‐cm H_2_O IVP with intravenous injection of PD0325901 (5 mg kg^−1^) 10 min before application of high IVP (right lane). *P <* *0.05, **P <* *0.01 compared to the control by a paired Student's *t*‐test.

After setting up the in vivo imaging of the bladder, we attempted to visualize the response of the urothelium under bladder distention with increased IVP, which often occurs during obstructive voiding dysfunction. According to the micturition pressure in mice with bladder outlet obstruction (Pandita et al. 2000), intermittent 100‐cm H_2_O IVP was applied for 1 min at 9‐min intervals to the transgenic mice expressing EKAREV‐NLS (Eisuke‐NLS‐FVB mice; Fig. 3A–C and Video S3). In all three experiments, the first 100‐cm H_2_O IVP overload increased ERK activity to the zenith within 10 min (Fig. 3C). The ERK activity declined to the basal level 1 h after the last IVP overload (Fig. 3C). An MEK inhibitor, PD0325901, abrogated the high IVP‐induced ERK activation (Fig. 3B and D). Next, to mimic the bladder with urinary retention, 60‐cm H_2_O IVP was applied for 30 min (Fig. 3E–G). To examine the generality of the high IVP‐induced ERK activation, we used two mouse strains, FVB/N and C57BL/6N. Both in mice with an FVB/N (Fig. 3E and F) and those with a C57BL/6N background (Fig. 3E and G), the continuous IVP overload induced high ERK activity in the urothelium, which remained during the high IVP. Intriguingly, ERK activity in the smooth muscle cells was not changed by the high IVP (Fig. 3E). We failed to detect ERK activation by 20‐ or 40‐cm H_2_O IVP, suggesting that the threshold was between 40‐ and 60 cm H_2_O (Fig. 3H). To validate the observations obtained by FRET imaging, the urothelium was isolated and analyzed by western blotting with an anti‐phospho‐ERK antibody, which showed elevated phospho‐ERK in the urothelium upon IVP stimulation (Fig. 3I). Because we did not find a significant difference between FVB/N and C57BL/6N, we used Eisuke‐NLS mice with a C57BL/6N background in the following experiments.

### Neither ischemia nor hydrostatic pressure is sufficient for ERK activation

**Figure 4:**
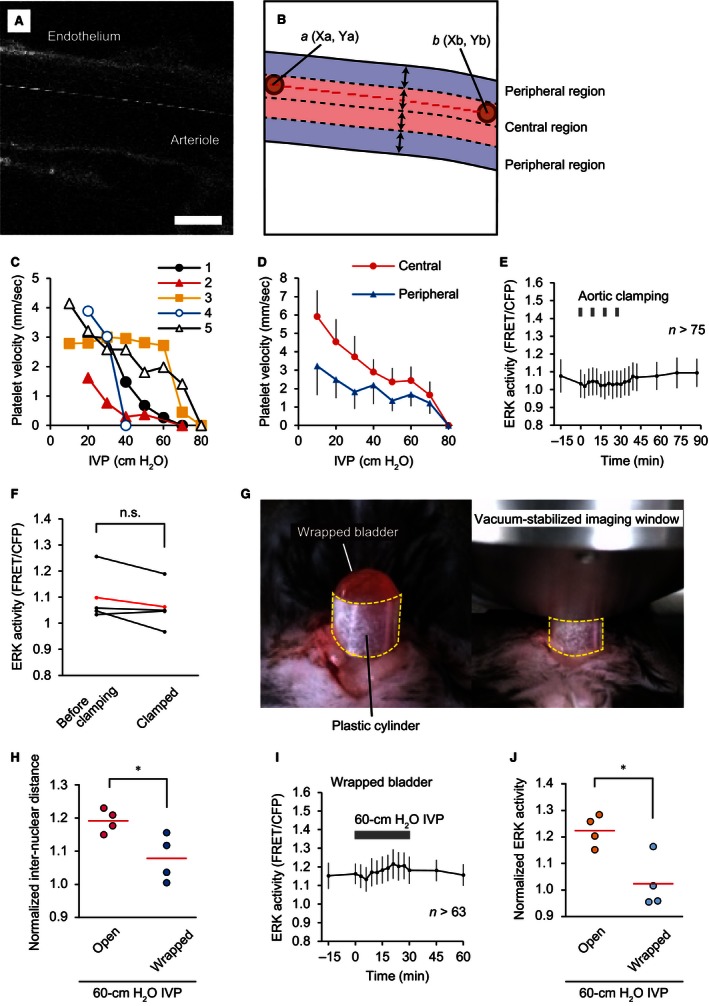
Exclusion of ischemia and hydrostatic pressure as potential causes of high intravesical pressure (IVP)‐induced extracellular signal‐regulated kinase (ERK) activation in the urothelium. (A and B) A representative image (A) and schematic diagram (B) of stroboscopic imaging of platelets in a suburothelial arteriole (Video S4). The lumen of the arteriole in each image is divided into the central region and peripheral region by the borderlines set at a fourth of the diameter from the surface of both sides of the endothelium. The platelet moves from the coordinates *a* (*Xa*,* Ya*) to *b* (*Xb*,* Yb*) in the time difference between the line scans. Scale bar = 10 *μ*m. (C) Relationship between IVP and platelet velocity in the suburothelial arterioles, as analyzed by stroboscopic measurement (*n *=* *5). The mean values of each experiment are shown. (D) Representative average platelet velocities in the central and peripheral regions from experiment no. 5. (E) A representative graph of the change in urothelial ERK activity by intermittent 1‐min aortic cross clamping every 9 min, for a total four rounds of cross clamping. *n*, number of cells analyzed at each time point. (F) Summary of measured ERK activity in the urothelium at baseline (0 min) and 9 min after the first round of aortic cross clamping (*n *=* *4). The ERK activity in each experiment and the mean values are indicated in black and red, respectively. n.s., not significant by a paired Student's *t*‐test. (G) Photographs showing the method of hampering bladder distention by high IVP. The mouse bladder was tightly wrapped with a plastic cylinder to prevent further distention by high IVP. Then, the vacuum‐stabilized imaging window was placed on the dome of the bladder. (H) Summary of the relative change in internuclear distance of the urothelial cells between baseline (0 min) and 30 min after application of 60‐cm H_2_O IVP to the bladder in an open space and the wrapped bladder (*n *=* *4 for each). Circles and red lines indicate the normalized internuclear distance of urothelial cells in each experiment and the mean values, respectively. (I) A representative graph of the change in urothelial ERK activity of the wrapped bladder by 30‐min 60‐cm H_2_O IVP. *n*, number of cells analyzed at each time point. (J) Summary of the relative change in urothelial ERK activity between baseline (0 min) and 30 min after application of 60‐cm H_2_O IVP to the bladder in an open space and the wrapped bladder (*n *=* *4 for each). Circles and red lines indicate normalized ERK activity in each experiment and the mean values, respectively. *P <* *0.05 compared to the control by an unpaired Student's *t*‐test.

In addition to stretch, the high IVP could affect at least two other physical parameters of the bladder: oxygen concentration and hydrostatic pressure. First, to assess the effect of IVP on oxygen concentration, we monitored the blood flow within the suburothelium. For this purpose, we developed a simple method to measure platelet velocity in the suburothelial arterioles. In Eisuke‐NES mice, platelets (ca. 2–3 *μ*m in diameter) could be distinguished from granulocytes (ca. 12–15 *μ*m in diameter) and lymphocytes (ca. 7 *μ*m in diameter) by their size. Red blood cells were also eliminated because these cells did not express the FRET biosensor. Due to the blood flow, the track of a single platelet was detected as a dashed line in a single scan image (Fig. 4A and B, and Video S4). From the coordinates of the platelet and the scan speed, the platelet velocity could be calculated as described in the Methods section. The initial platelet velocity under 10‐ or 20‐cm H_2_O IVP was 3.1 ± 1.0 mm sec^−1^ (Fig. 4C). With increasing IVP, the platelet velocities gradually decreased and reached zero at 68 ± 16‐cm H_2_O IVP (Fig. 4C). The mean platelet velocities in the central regions were higher than those in the peripheral regions (Fig. 4D). Therefore, high IVP (60 and 100 cm H_2_O), which was sufficient to activate ERK in the urothelium, should cause more or less ischemia in the urothelium. This finding prompted us to examine whether ischemia activated ERK in the urothelium. For this purpose, we repeatedly clamped and declamped the distal end of the abdominal aorta during imaging to recapitulate the condition of Figure 3C. Although blood flow was halted, no ERK activity changes were observed by this procedure (Fig. 4E and F), negating the possibility that the ischemia under high IVP caused ERK activation.

Second, we examined whether increased hydrostatic pressure activated ERK. To exclude the effect of increased tension or stretch under 60‐cm H_2_O IVP, bladder distention was minimized by wrapping the bladder with a plastic cylinder (Fig. 4G). The urothelial extension under 60‐cm H_2_O IVP was monitored by the distances between nuclei. The 60‐cm H_2_O IVP increased the internuclear distance up to 19% (Fig. 4H). The plastic cylinder reduced the increase in internuclear distance to 8% (Fig. 4H), and almost completely inhibited the high IVP‐induced ERK activation (Fig. 4I and J). Taken together, these results indicate that neither ischemia nor increased hydrostatic pressure could cause ERK activation in the urothelium, leaving the stretch as the potential cause.

### Stretching of TRT‐HU1 urothelial cells activates ERK

**Figure 5:**
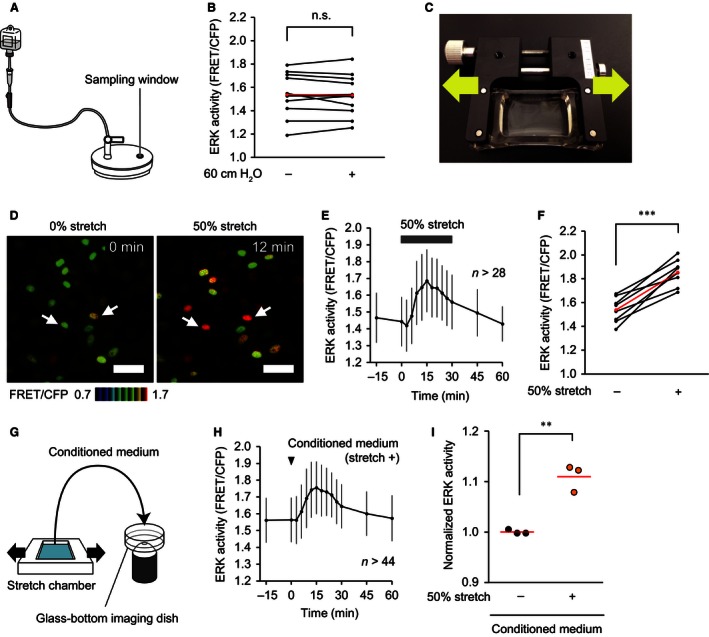
Extracellular signal‐regulated kinase (ERK) activation by stretch‐evoked release of specific molecules in TRT‐HU1 cells. (A) A schematic diagram of the handmade device used to apply hydrostatic pressure to TRT‐HU1 cells. (B) Summary of measured ERK activity in TRT‐HU1 cells at baseline (0 min) and 30 min after application of 60‐cm H_2_O hydrostatic pressure (*n *=* *12). The ERK activity in each experiment and the mean values are indicated in black and red, respectively. n.s., not significant by a paired Student's *t*‐test. (C) A photograph of the uniaxial stretch experiment. A silicon chamber containing confluent TRT‐HU1 cells was uniaxially stretched with a manual extension device. (D) Representative ratio images of stretch‐evoked ERK activation in TRT‐HU1 cells (Video S5). Two representative cells are marked (arrows). Scale bar = 50 *μ*m. (E and F) A representative graph (E) and summary (F) of the change in ERK activity of TRT‐HU cells by 30‐min 50% uniaxial stretch (*n *=* *8). *n*, number of cells analyzed at each time point (E). ERK activity was measured at baseline (0 min) and 15 min after application of uniaxial stretch (F). ERK activity in each experiment and the mean values are indicated in black and red, respectively (F). (G) A schematic diagram of the medium treatment experiment. During imaging of TRT‐HU1 cells, the other TRT‐HU1 cells were treated with 50% uniaxial stretch for 1 min. The culture medium of the cells under observation was removed, and replaced with the conditioned medium of the stretched cells. (H) A representative graph of the change in ERK activity of TRT‐HU1 cells by treatment with the conditioned medium of the stretched cells. *n*, number of cells analyzed at each time point. (I) Summary of the relative change in ERK activity of TRT‐HU1 cells between baseline (0 min) and 15 min after addition of conditioned medium of the stretched cells and nonstretched cells (*n *=* *3 for each). Circles and red lines indicate normalized ERK activity in each experiment and the mean values, respectively. **P <* *0.01, ***P <* *0.001 compared to the control by a paired Student's *t*‐test.

We attempted to set up in vivo experiments to induce urothelial stretch without affecting blood flow or hydrostatic pressure; however, technical difficulties impeded the successful intravital monitoring of urothelial ERK activity under urothelial stretch. Therefore, we used human immortalized urothelial cells, TRT‐HU1 cells, to investigate whether hydrostatic pressure or stretch activated ERK. We made a device to apply hydrostatic pressure to TRT‐HU1 cells during time‐lapse imaging (Fig. 5A). We applied a 30‐min 60‐cm H_2_O hydrostatic pressure to TRT‐HU1 cells, and confirmed that the hydrostatic pressure did not activate ERK (Fig. 5B). Finally, to examine the last possibility – that mechanical stretch activates ERK activity in the urothelium – we applied uniaxial stretch to the TRT‐HU1 cells plated on elastic silicone chambers (Fig. 5C), and found that 50% stretch increased ERK activity within 15 min (Fig. 5D–F, and Video S5). Surprisingly, addition of the conditioned medium of the stretched cells activated ERK in nonstretched cells (Fig. 5G–I), suggesting the involvement of certain molecules released from the stretched cells in the stretch‐evoked ERK activation.

### ATP activates ERK in the stretched TRT‐HU1 cells

**Figure 6:**
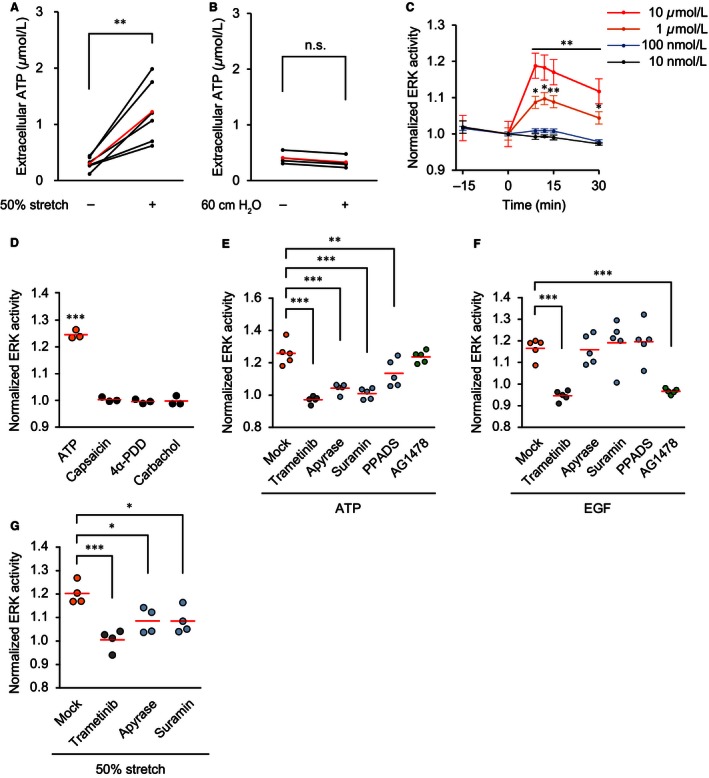
Extracellular signal‐regulated kinase (ERK) activation by autocrine/paracrine ATP signaling in stretched TRT‐HU1 cells. (A and B) ATP concentration in the culture medium of TRT‐HU1 cells before and after application of 1 min of 50% uniaxial stretch (*n *=* *6) (A) and 5 min of 60‐cm H_2_O hydrostatic pressure (*n *=* *4) (B). The ATP concentration in each sample and the mean values are indicated in black and red, respectively. **P <* *0.01 compared to the control by a paired Student's *t*‐test. n.s., not significant by a paired Student's *t*‐test. (C) Summary of the relative change in ERK activity of TRT‐HU1 cells by treatment with ATP (10 nmol L^−1^–10 *μ*mol L^−1^) (*n *=* *5 for each). The FRET/CFP ratio is normalized at time 0. *P <* *0.05, **P <* *0.01 compared to the control by a paired Student's *t*‐test. (D) Summary of the relative change in ERK activity of TRT‐HU1 cells by treatment with ATP (10 *μ*mol L^−1^), capsaicin (10 *μ*mol L^−1^), 4*α*‐PDD (10 *μ*mol L^−1^) and carbachol (10 *μ*mol L^−1^) between baseline (0 min) and 9–12 min after addition of the drugs (*n *=* *4 for each). ***P <* *0.001 compared to the control by a paired Student's *t*‐test. (E and F) Summary of the relative change in ERK activity of TRT‐HU1 cells by the effects of pretreatment with trametinib (100 nmol L^−1^), apyrase (4 U mL ^−1^), suramin (200 *μ*mol L^−1^), PPADS (200 *μ*mol L^−1^) and AG1478 (10 *μ*mol L^−1^) on ATP‐induced ERK activation (*n *=* *5 for each) (E) and EGF‐induced ERK activation (*n *=* *5 for each) (F). ATP (10 *μ*mol L^−1^) and EGF (10 ng mL ^−1^) were added 9 min after pretreatment with the inhibitory drugs. (G) Summary of the relative change in ERK activity of TRT‐HU1 cells by the effects of pretreatment with trametinib (100 nmol L^−1^), apyrase (4 U mL ^−1^), and suramin (200 *μ*mol L^−1^) on stretch‐evoked ERK activation (*n *=* *4 for each). A 30‐min 50% uniaxial stretch was applied 9 min after pretreatment with the inhibitory drugs. ERK activity was measured at baseline (0 min) and 9–15 min after addition of ATP (E) and EGF (F), and application of stretch (G). Circles and red lines indicate normalized ERK activity in each experiment and the mean values, respectively (D–G). *P <* *0.05, **P <* *0.01, ***P <* *0.001 compared to the control by one‐way ANOVA with post hoc Dunnett's test (E–G).

Urothelial stretch has been shown to evoke ATP release (Birder et al. 2012). In agreement with the previous report, we found that 50% stretch increased the ATP concentration to 1.2 ± 0.6 *μ*mol L^−1^ (Fig. 6A). On the other hand, the 60‐cm H_2_O hydrostatic pressure did not cause ATP release into the culture medium (Fig. 6B). In TRT‐HU1 cells, ATP‐activated ERK in a dose‐dependent manner (Fig. 6C). Next, the contribution of other mechanosensors was examined using the transient receptor potential vanilloid (TRPV) 1 activator capsaicin, the TRPV4 activator 4*α*‐PDD, and the muscarinic receptor activator carbachol. None of them activated ERK in TRT‐HU1 cells (Fig. 6D). Moreover, in addition to the MEK inhibitor trametinib (100 nmol L^−1^), the ATP diphosphohydrolase apyrase (4 U mL^−1^) and the nonselective P2 receptor inhibitor suramin (200 *μ*mol L^−1^) completely suppressed ATP‐induced ERK activation, whereas the nonselective P2X receptor inhibitor PPADS (200 *μ*mol L^−1^) partially suppressed the ATP‐induced ERK activation (Fig. 6E). To rule out the possibility that ATP‐evoked ERK activation was mediated by transactivation of the EGF receptor by ATP, we examined the effects of the EGF receptor inhibitor AG1478 (10 *μ*mol L^−1^) on ATP‐evoked ERK activation, but could not detect any inhibition (Fig. 6E). The specificity of the inhibitors was confirmed by EGF stimulation: none of the inhibitors of ATP signaling – that is, apyrase, suramin, or PPADS – suppressed the EGF‐induced ERK activation, whereas trametinib and AG1478 did completely suppress the ERK activation (Fig. 6F). Finally, we confirmed that stretch‐evoked ERK activation was dependent on autocrine/paracrine ATP signaling. As shown in Figure 6G, both apyrase and suramin suppressed stretch‐evoked ERK activation. Thus, ATP‐mediated activation of P2X and P2Y receptors appeared to be the primary cause of ERK activation by the stretch of TRT‐HU1 cells.

### ATP mediates urothelial stretch‐induced ERK activation in vivo

**Figure 7:**
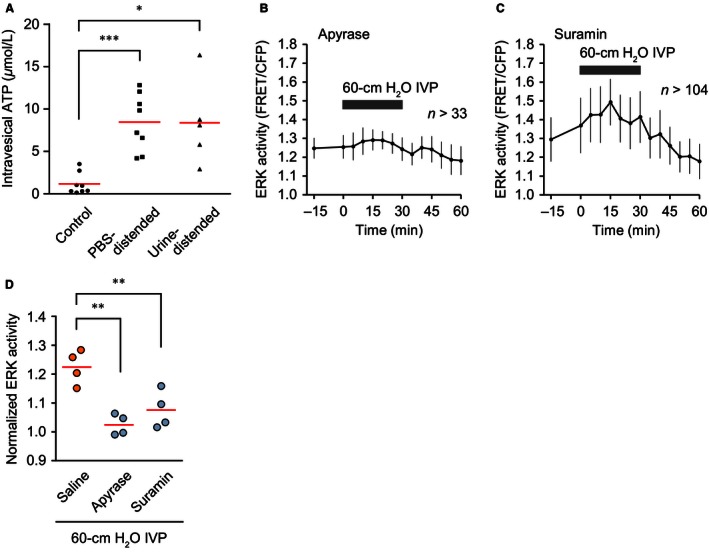
High intravesical pressure (IVP)‐induced extracellular signal‐regulated kinase (ERK) activation by autocrine/paracrine ATP signaling in the urothelium of live mice. (A) ATP concentration in the urine in the storage phase (*n *=* *8) and the samples from PBS‐distended bladders (*n *=* *8) and urine‐distended bladders (*n *=* *5). Black dots and red lines indicate the ATP concentration in each sample and the mean values, respectively. *P <* *0.05, ***P <* *0.001 compared to the control by an unpaired Student's *t*‐test. (B and C) Representative graphs of the change in urothelial ERK activity by the effects of pretreatment with apyrase (5 U, 100 *μ*L) (B) and suramin (2 mmol L^−1^, 100 *μ*L) (C) on ERK activation by 30‐min 60‐cm H_2_O IVP. *n*, number of cells analyzed at each time point. (D) Summary of the relative change in urothelial ERK activity between baseline (0 min) and 30 min after application of 60‐cm H_2_O IVP with or without pretreatment with apyrase and suramin (*n *=* *4 for each). Circles and red lines indicate normalized ERK activity in each experiment and the mean values, respectively. **P <* *0.01 compared to the control by one‐way ANOVA with post hoc Dunnett's test.

To examine whether ATP mediates ERK activation by high IVP‐induced urothelial stretch in vivo, we measured ATP concentration in the bladder. For control samples, the urine was collected in the urine storage phase. For samples of the PBS‐distended bladder, 150 *μ*L PBS (37°C) was infused into the empty bladder. To exclude the possibility that PBS triggers ATP secretion, in a third set of samples, urine was collected and returned to the bladder to induce distension. One minute later, the PBS or the returned urine was recovered to monitor ATP concentration. As expected, ATP concentrations in both the PBS‐ and urine‐distended bladder were increased to 8.5 ± 3.3 and 8.4 ± 5.0 *μ*mol L^−1^, respectively, in comparison to 1.2 ± 1.3 *μ*mol L^−1^ in the control urine (Fig. 7A). Next, we investigated whether or not apyrase and suramin inhibited the high IVP‐induced ERK activation. For this purpose, we emptied the bladder, and infused 100 *μ*L apyrase (5 U) or suramin (2 mmol L^−1^) through the urethral catheter. Both apyrase and suramin inhibited the high IVP‐induced ERK activation (Fig. 7B–D). Thus, we concluded that high IVP induced ATP secretion, which in turn activated ERK in the urothelium in an autocrine/paracrine manner through the activation of P2 receptors.

## Discussion

ERK, which plays an important role in cellular proliferation and regeneration in many cell types, including cells of the urothelium (Swiatkowski 2003; Ling et al. 2011), can also transmit extracellular signals triggered by, for example, ischemia‐reperfusion (Seko et al. 1996), compressive stress (Tschumperlin et al. 2002), and mechanical stretch (Sadoshima and Izumo 1993; Balestreire and Apodaca 2007). This property renders the mice expressing a FRET biosensor for ERK an excellent research tool to examine the in vivo cellular response induced by extracellular stimuli.

On the other hand, the in vivo imaging cannot associate extracellular stimuli directly with a given output – in our case, ERK activation. In fact, in addition to stretch, elevated IVP could affect urothelial ERK activity by at least two different mechanisms: ischemia and elevated hydrostatic pressure. We first examined whether elevated IVP causes ischemia. Bladder blood flow has been monitored by laser Doppler blood flowmetry (Wardell et al. 1993; Greenland and Brading 1996; Okutsu et al. 2011), or by using pencil lens charge‐coupled device microscopy systems (Mizuno et al. 2010; Mine et al. 2013). We took advantage of 2PM, which visualizes platelet flow. Previous studies have investigated platelet velocities in the pulmonary and mesenteric arterioles of mice, demonstrating that the platelet velocities ranging from 0.5 to 3.5 mm sec^−1^ (Tangelder et al. 1986; Eichhorn et al. 2002). We obtained similar velocities of between 1.6 to 4.1 mm sec^−1^ in the suburothelial arterioles under 10–20‐cm H_2_O IVP, and also observed that the blood flow was stopped or largely decreased under elevated IVP conditions (60 and 100 cm H_2_O), raising the possibility that ischemia may cause ERK activation in the urothelium (Fig. 4). This possibility was negated by the transient aortic clamping experiment, which did not cause ERK activation in the urothelium.

Previous in vitro studies have shown that the urothelium could sense hydrostatic pressure changes of 5–20 cm H_2_O and secrete ATP (Ferguson et al. 1997; Wang et al. 2005; Olsen et al. 2011). However, when the mechanical stretch was minimized by enclosing the bladder in a plastic cylinder, elevated IVP failed to activate ERK in the urothelium (Fig. 4), implying that elevated hydrostatic pressure is not sufficient for urothelial ERK activation in vivo. Moreover, using TRT‐HU1‐immortalized urothelial cells, we found that increase in hydrostatic pressure could not activate ERK (Fig. 5).

On the other hand, mechanical stretch clearly activated ERK in an ATP‐dependent manner (Fig. 6). This raises a question: What are the roles of ATP‐dependent ERK activation in the urothelium? It has been reported that stretch of the urothelium promotes exocytosis of fusiform vesicles to increase the cell surface through activation of ERK (Balestreire and Apodaca 2007). This report describes that ERK activation is regulated by autocrine activation of apical EGF receptors by heparin‐binding EGF. In agreement with this observation, we have shown that ERK activation activates metalloproteases and thereby promotes cleavage of pro‐EGF ligands (Aoki et al. 2013b; Hiratsuka et al. 2015). However, at least within TRT‐HU1 cells, the EGF receptor inhibitor AG1478 failed to suppress ATP‐induced ERK activation. It should be further examined whether the EGF receptor is involved in the ERK activation induced by increased IVP.

Lastly, we should admit that there were several technical limitations in the present in vivo imaging of the bladder. First, only female mice could be used, because the longer urethra makes it impossible to insert the urethral catheter into the bladder of male mice. Second, physiological spontaneous rhythmic contraction of the detrusor smooth muscle hampers stable time‐lapse imaging (Video S1). Third, the bladder neck could not be observed due to the design of the vacuum‐stabilized imaging window. Finally, imaging of the urothelium requires 15–20‐cm H_2_O IVP, which is slightly above the normal IVP in the urine storage phase. Nevertheless, in vivo imaging of the bladder would potentially be applicable to investigations into the microstructure, hemodynamics, urodynamics, and molecular dynamics of the mouse bladder.

## Conflict of Interest

There are no competing interests for any of the authors.

## Supporting information




**Video S1.** Merged z‐stack images of CFP, SHG, and Qtracker 655 of the whole mouse bladder wall expressing EKAREV‐NES from the urothelium to the muscle layer.

**Video S2.** Merged z‐stack images of CFP, SHG, and Qtracker 655 of the whole mouse bladder wall expressing EKAREV‐NLS from the urothelium to the muscle layer.

**Video S3.** ERK activation in the urothelium of an Eisuke‐NLS‐FVB mouse by intermittent 1‐min 100‐cm H_2_O IVP.

**Video S4.** Stroboscopic imaging of platelets in a suburothelial arteriole.

**Video S5.** Stretch‐evoked ERK activation in TRT‐HU1 cells.
