# Supplementary material for: Intravital imaging of mouse urothelium reveals activation of extracellular signal‐regulated kinase by stretch‐induced intravesical release of ATP
Source: Physiol Rep. 2016 Nov 15;4(21):e13033. doi: 10.14814/phy2.13033 (PMC5112504; doi:10.14814/phy2.13033)
Supplement: Supplementary file 6 [file PHY2-4-e13033-s006.docx]

**Video S1.** Merged z-stack images of CFP, SHG and Qtracker 655 of the whole mouse bladder wall expressing EKAREV-NES from the urothelium to the muscle layer.

**Video S2.** Merged z-stack images of CFP, SHG and Qtracker 655 of the whole mouse bladder wall expressing EKAREV-NLS from the urothelium to the muscle layer.

**Video S3.** ERK activation in the urothelium of an Eisuke-NLS-FVB mouse by intermittent 1-min 100-cm H_2_O IVP.

**Video S4.** Stroboscopic imaging of platelets in a suburothelial arteriole.

**Video S5.** Stretch-evoked ERK activation in TRT-HU1 cells.
